# Supplementary material for: The genome sequence of the Moorland Grey, Eudonia murana (Curtis, 1827) (Lepidoptera: Crambidae)
Source: Wellcome Open Res. 2025 Dec 22;10:686. [Version 1] doi: 10.12688/wellcomeopenres.25207.1 (PMC12946702; doi:10.12688/wellcomeopenres.25207.1)
Supplement: Peer review history

# Reviewer report: Reviewer response for version 1

**DOI:** 10.21956/wellcomeopenres.27783.r148701

**Published:** 2026-02-26

**Authors:** Danilo Trabudo do Amaral

**Affiliations:** 1 Universidade Federal do ABC Centro de Ciencias Naturais e Humanas (Ringgold ID: 425753), Santo André, State of São Paulo, Brazil

This study presents a high-quality, chromosome-level genome assembly of *Eudonia murana*, generated using state-of-the-art long-read and Hi-C technologies. The work represents an important contribution to the Darwin Tree of Life initiative by providing a reliable genomic resource for future ecological, evolutionary, and comparative studies. The methodology is robust, and the data are well documented and openly accessible.

 As minor suggestions, the authors could strengthen the manuscript by including a brief comparison with closely related species, adding a short discussion on the evolutionary implications of the Z1/Z2/W sex chromosome system, and clarifying the differences in completeness between the two haplotypes.

Are sufficient details of methods and materials provided to allow replication by others?

Yes

Is the rationale for creating the dataset(s) clearly described?

Yes

Are the datasets clearly presented in a useable and accessible format?

Yes

Are the protocols appropriate and is the work technically sound?

Yes

Reviewer Expertise:

Bioinformatics

I confirm that I have read this submission and believe that I have an appropriate level of expertise to confirm that it is of an acceptable scientific standard.

# Reviewer report: Reviewer response for version 1

**DOI:** 10.21956/wellcomeopenres.27783.r146697

**Published:** 2026-02-26

**Authors:** Marko Mutanen

**Affiliations:** 1 University of Oulu, Oulu, Finland

This study presents the genome sequence for a crambid moth Eudonia murana. This species is a member of a taxonomically difficult group of species, but as far as is known, DNA barcode provides a safe way to distinguish this species from all of its close relatives. It is therefore good that the identification was confirmed by barcoding. Interestingly, the samples specimens undoubtedly represent a second-generation specimen; a rare thing in this group of species but known for E. murana. Unlike stated in the introduction, this species is readily separate from Scoparia spp. in genitalia (dissection). It is very difficult to tell apart from E. truncicolella by genital characteristics.

 The genome sequence is clearly of high quality and will serve as an excellent reference genome for the species. The snail plot figure, for example, demonstrates this high quality. For example, the BUSCO recovery rate is high. I have not reservations about the quality of the genome sequence.

Are sufficient details of methods and materials provided to allow replication by others?

Yes

Is the rationale for creating the dataset(s) clearly described?

Yes

Are the datasets clearly presented in a useable and accessible format?

Yes

Are the protocols appropriate and is the work technically sound?

Yes

Reviewer Expertise:

Lepidoptera diversity, molecular taxonomy, sawfly diversity, DNA barcoding, phylogenetics

I confirm that I have read this submission and believe that I have an appropriate level of expertise to confirm that it is of an acceptable scientific standard.
